# Bottlenecks can constrain and channel evolutionary paths

**DOI:** 10.1093/genetics/iyad001

**Published:** 2023-02-02

**Authors:** Jasmine Gamblin, Sylvain Gandon, François Blanquart, Amaury Lambert

**Affiliations:** Center for Interdisciplinary Research in Biology (CIRB), College de France, CNRS, INSERM, Université PSL, 75005 Paris, France; Centre d’Ecologie Fonctionnelle et Evolutive (CEFE), CNRS, Univ Montpellier, EPHE, IRD, 34293 Montpellier, France; Center for Interdisciplinary Research in Biology (CIRB), College de France, CNRS, INSERM, Université PSL, 75005 Paris, France; Infection, Antimicrobials, Modelling, Evolution (IAME), INSERM, Université Paris Cité, 75870 Paris, France; Center for Interdisciplinary Research in Biology (CIRB), College de France, CNRS, INSERM, Université PSL, 75005 Paris, France; Institut de Biologie de l’ENS (IBENS), École Normale Supérieure (ENS), CNRS, INSERM, Université PSL, 75005 Paris, France

**Keywords:** forecasting evolution, demography, microbial evolution, stochastic model, adaptation

## Abstract

Population bottlenecks are commonplace in experimental evolution, specifically in serial passaging experiments where microbial populations alternate between growth and dilution. Natural populations also experience such fluctuations caused by seasonality, resource limitation, or host-to-host transmission for pathogens. Yet, how unlimited growth with periodic bottlenecks influence the adaptation of populations is not fully understood. Here, we study theoretically the effects of bottlenecks on the accessibility of evolutionary paths and on the rate of evolution. We model an asexual population evolving on a minimal fitness landscape consisting of two types of beneficial mutations with the empirically supported trade-off between mutation rate and fitness advantage, in the regime where multiple beneficial mutations may segregate simultaneously. In the limit of large population sizes and small mutation rates, we show the existence of a unique most likely evolutionary scenario, determined by the size of the wild-type population at the beginning and at the end of each cycle. These two key demographic parameters determine which adaptive paths may be taken by the evolving population by controlling the supply of mutants during growth and the loss of mutants at the bottleneck. We do not only show that bottlenecks act as a deterministic control of evolutionary paths but also that each possible evolutionary scenario can be forced to occur by tuning demographic parameters. This work unveils the effects of demography on adaptation of periodically bottlenecked populations and can guide the design of evolution experiments.

## Introduction

Population bottlenecks are sudden, drastic reductions of population size that can arise both in vivo and in vitro. Pathogen populations experience such bottlenecks during host to host transmission (Abel *et al.* 2015; Geoghegan *et al.* 2016), or they can be induced by resource limitation or seasonality (e.g. the boom and bust dynamics of phytoplankton; Behrenfeld *et al.* 2017). They also are commonplace in experimental evolution: in serial passaging (or transfer) experiments, a microbial population is periodically subsampled and placed on new medium to grow again (Kawecki *et al.* 2012). In this way, microbial populations can be followed during several generations (Good *et al.* 2017) while remaining of a manageable size.

Several studies have investigated how periodic bottlenecks influence the rate of adaptation of such populations. They have mostly focused on the probability of stochastically losing beneficial mutations (Wahl and Gerrish 2001), on the time of arrival of successful mutations (Wahl *et al.* 2002), on mutant fixation (Hall *et al.* 2010; Garoff *et al.* 2020; Schenk *et al.* 2022), and on the predictability of evolution (Szendro *et al.* 2013; Freitas *et al.* 2021) with applications to the study of drug resistance (Huseby *et al.* 2017; Nicholson and Antal 2019; Mahrt *et al.* 2021) (see LeClair and Wahl 2018 for a review).

Here we examine theoretically how demography affects not only the rate of adaptation but also the evolutionary paths followed by bottlenecked populations. In populations with constant size evolving in a regime of strong selection-strong mutation (as is often the case for experimental asexual populations), the distribution of fitness effect of fixed mutations and the rate of adaptation are dictated by the population size, the mutation rate, and the shape of the distribution of fitness effects (Desai and Fisher 2007; Fogle *et al.* 2008). In bottlenecked populations, both the supply of beneficial mutations and the probability of establishment change over time, and the accessibility of evolutionary paths, can in theory depend on bottleneck size or severity and on cycle duration. Furthermore, adaptation may increase population size, feed back on the mutation supply, and enhance the scope for further adaptation. These effects are potentially important for experimental and natural evolution but have not been studied theoretically.

To study how bottlenecks influence the rate and paths of adaptation, we assume a minimal fitness landscape with a trade-off between the rate of appearance of beneficial mutations and their fitness advantage, consistently with the decreasing distribution of fitness effects documented in multiple species (Eyre-Walker and Keightley 2007). We take advantage of large population limit techniques as is standard in population genetics, notably in Luria–Delbrück type fluctuation experiments of microbial populations (Luria and Delbrück 1943; Zheng 1999). We study the timing of emergence and establishment of beneficial mutations to investigate the effect of bottleneck size and cycle duration, or equivalently of the initial and final population sizes of the wild-type (WT), on which paths are accessible to evolution.

## Model

**Fig. 1.**
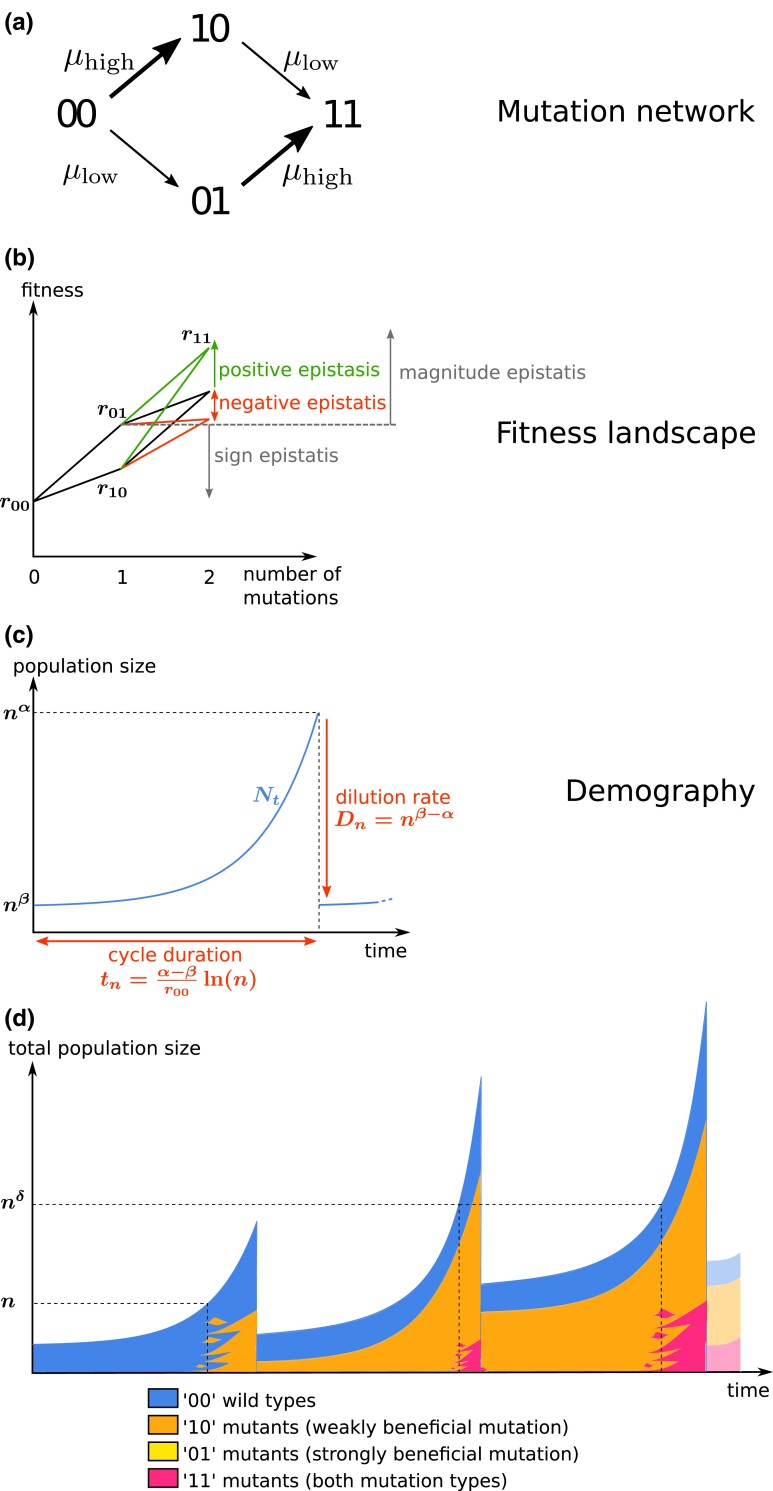
a) Mutation network. b) Fitness landscape, growth rate as a function of the number of mutations. c) Demography during one cycle of growth, WT population size as a function of time. d) Illustration of demography during three cycles of growth, total population size as a function of time. Mutants start to appear when the population size is around the inverse of the mutation rate. Mutant subpopulations are composed of multiple independent clones, which can go extinct due to stochasticity (genetic drift, bottleneck). The illustrated scenario corresponds to the blue bottom-right area in Fig. 2, where strongly beneficial “01” mutants never establish in the population but weakly beneficial “10” mutants and double mutants do.

We consider an asexual population adapting to a new environment. We assume two types of beneficial mutations, with a trade-off between fitness and mutation rate. High-rate, weakly beneficial mutations confer a moderate gain in fitness, while low-rate, strongly beneficial mutations confer a large gain in fitness. This setting can be thought of as a coarse discretization of a decreasing (e.g. exponential) distribution of fitness effects (Kassen and Bataillon 2006). These two types of mutations can be thought to target different loci underlying traits linked to adaptation, e.g. resistance to a drug or predator, or the exploitation of a new resource. We thus obtain a simple fitness landscape composed of four genotypes: “00” for the WT, “10” for individuals with a weakly beneficial mutation, “01” for individuals with a strongly beneficial mutation, and “11” for individuals carrying both types of mutation. Thus, mutations 00⟶10 and 01⟶11 are weakly beneficial, while 00⟶01 and 10⟶11 are strongly beneficial. We allow magnitude epistasis but not sign epistasis, thus the growth rates of the four genotypes verify: $r_{11}>r_{01}>r_{10}>r_{00}$ (see Fig. 1b). We neglect the production of double mutants by recombination between single mutants.

### A semi-deterministic model

Population size grows exponentially and is subject to periodic bottlenecks of fixed relative severity, i.e. the fraction of population that survives is constant (as opposed to fixed absolute bottleneck severity, where the number of individuals that survive is constant; LeClair and Wahl 2018). As a consequence, the WT population goes back to its initial size $N_{0}$ at the beginning of each cycle. However, the size of the whole population can increase through successive transfers due to the arrival of beneficial mutants, see Fig. 1d. We use a semi-deterministic model to describe the dynamics of the population. We assume that $N_{0}$ is sufficiently large that the growth of the WT population during one cycle can be described deterministically: $N_{t}=N_{0}e^{r_{00}t}$. In contrast, the population dynamics of mutants, always starting in small numbers of copies, will be described by a stochastic birth-death model. The weakly beneficial and strongly beneficial mutation rates are, respectively, $\mu_{\mathrm{high}}$ and $\mu_{\mathrm{low}}$, with $\mu_{\mathrm{high}}\gg \mu_{\mathrm{low}}$ (see Fig. 1a). We denote by $\mu =\mu_{\mathrm{high}}+\mu_{\mathrm{low}}$ the total mutation rate to beneficial mutations. This semi-deterministic setting is similar to the one first used in Lea and Coulson (1949) to model the Luria–Delbrück experiment, and more recently in Keller and Antal (2015).

### Large population, small mutation rate assumption

The mutation rate to a beneficial mutation is typically very small, while the size of microbial populations is usually quite large. Thus we introduce a scaling parameter n that is of the order of 1/μ, and we will assume in the following that n≫1. In the large n limit the probability of most events of interest approaches either 1 or 0, allowing us to determine the most likely scenario in a given parameter setting. To comply with $\mu_{\mathrm{high}}\gg \mu_{\mathrm{low}}$, we also introduce a parameter δ>1 such that


$$\mu_{\mathrm{high}}=\frac{1}{n}\;\text{and}\;\mu_{\mathrm{low}}=\frac{1}{n^{\delta }}$$

We suppose that the time $t_{n}$ between two dilutions is such that during one cycle of the experiment, the WT population grows from a size $N_{0}=n^{\beta }$ to $n^{\alpha }$. As a consequence,


$$t_{n}=(\alpha -\beta )\frac{\ln\;(n)}{r_{00}}$$

We constrain β to be in (0,1) and α to be greater than 1, in order to have $N_{0}\ll n\ll N_{t_{n}}$. In that way, we ensure that there is no mutant at the beginning of the experiment, and that weakly beneficial mutants will appear during the first cycle with high probability. Because of the large n assumption, there is a sharp transition between a regime where mutations are very unlikely to occur (for $N_{t}\mu \ll 1$) to a regime where numerous mutations arise (for $N_{t}\mu \gg 1$). Thus we expect to observe adaptation via multiple-origins soft sweeps in the second regime (Messer and Petrov 2013; Hermisson and Pennings 2017), in agreement with empirical observations in microbes (Nair *et al.* 2006; Barroso-Batista *et al.* 2014; Pennings *et al.* 2014). The dilution factor between two cycles must be chosen so that the WT population always starts afresh at the same size $N_{0}=n^{\beta }$. Thus the dilution factor is


$$D_{n}=\frac{1}{n^{\alpha -\beta }}$$

The notation is summarized in Fig. 1 and Table 1.

**Table 1.** Main parameters of the model.

Notation	Interpretation
$r_{00}$	Growth rate of WT
$r_{10},r_{01}$	Growth rates of single mutants
$r_{11}$	Growth rate of double mutant
$\mu_{\mathrm{high}}=1/n$	Weakly beneficial mutation rate
$\mu_{\mathrm{low}}=1/n^{\delta }$	Strongly beneficial mutation rate
$\mu =\mu_{\mathrm{high}}+\mu_{\mathrm{low}}$	Global beneficial mutation rate
$N_{t}$	Size of the WT population at time t
$N_{0}=n^{\beta }$	Initial WT population size
$N_{t_{n}}=n^{\alpha }$	Final WT population size
$t_{n}=(\alpha -\beta )\ln\;(n)/r_{00}$	Time duration of one cycle
$D_{n}=n^{\beta -\alpha }$	Dilution factor

## Results

Here we analyze the dynamics of adaptation by characterizing the evolutionary paths followed by evolution, the timing of this process, and how it depends on demographic parameters. For detailed derivations, see Supplementary File S1, which also provides insight into the timing of weakly beneficial mutations during the first cycle. When a mutant population arises at cycle k, escapes stochastic extinction, and so is present in non-negligible quantity at the end of the growth phase, we will say that this population has *established* during cycle k. If the established mutant population reaches a sufficiently large size that it survives the next bottleneck (and so every subsequent bottleneck), we will say that this subpopulation *survives*. We say that an event A occurs with high probability (w.h.p.) if P(A)→1 as n→+∞.

### Effect of demography on evolutionary paths

#### Single mutant “10” establishes w.h.p. at first cycle (assuming α>1)

The product of the WT population size at the end of first cycle and of the high mutation rate is $\mu_{\mathrm{high}}N_{t_{n}}=(1/n)n^{\alpha }\gg 1$. We rigorously showed in Supplementary File S1 that “10” mutants establish w.h.p. during the first cycle and computed an estimate of the number $Z_{10}^{\left(n\right)}$ of “10” mutants at the end of this cycle.

#### Single mutant “01” establishes w.h.p. at first cycle if α>δ (and if α<δ w.h.p. never arises)

The establishment of mutants “01” depends on the relative values of the final population size and the rate of strongly beneficial mutations, governed by parameters α and δ. If α>δ, then $\mu_{\mathrm{low}}N_{t_{n}}\gg 1$ and mutants “01” also establish during the first cycle. If on the contrary α<δ, the probability that mutants “01” arise in the first cycle is close to 0. As the WT population has exactly the same size at the end of each cycle, it is unlikely that mutants “01” establish in the course of the experiment. This highlights that the demographic control imposed on the WT population affects the establishment of mutations. In fact, it also affects which evolutionary paths are accessible: in the case where α<δ, the transition 00⟶01 is not possible (and neither is 01⟶11).

#### Significance of demographic parameters α and β

**Fig. 2.**
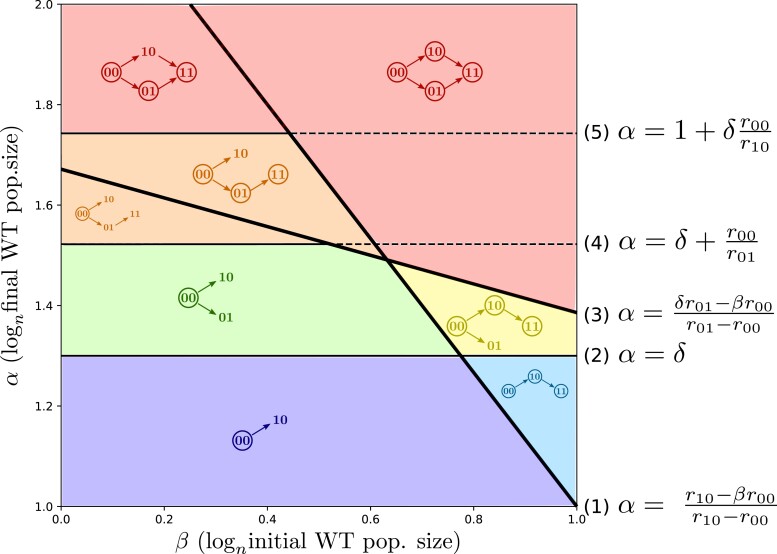
Predicted evolutionary paths, as a function of demographic parameters β and α. These predictions are made for infinitely large populations and vanishing mutation rates. Arrows are observed evolutionary paths. Genotypes that are shown but not circled are establishing but not surviving. Genotypes that are circled are surviving. The six colors (red, orange, yellow, green, blue, and purple) correspond to six different predicted path configurations. The dilution ratio is constant along lines of slope 1 (δ=1.3, $r_{00}=0.2$, $r_{10}=0.35$, and $r_{01}=0.9$).

For fixed values of the growth rates ($r_{00}$, $r_{10}$, and $r_{01}$) and of δ, we can project on the plane (β,α) areas corresponding to different configurations of evolutionary paths. All path configurations must include the 00⟶10 transition because “10” establish w.h.p. during the first cycle, thus there are six possible configurations. Figure 2 shows the areas corresponding to different path configurations for a chosen parameter set. This set was such that these six configurations are present, but this is not always the case. Equations for threshold lines (1–5) are derived and explained in Supplementary File S1. These equations are derived for n→+∞, i.e. for infinitely large populations and vanishing mutation rates. See Supplementary Fig. S2 from Supplementary File S1 for observed evolutionary paths in simulations with finite values of n. Together, α and β determine the initial and final WT population size at each cycle, the duration of each cycle, and the relative severity of each bottleneck. When α increases, the final WT population size, bottleneck severity, and cycle duration increase. When β increases, the initial WT population size increases but bottleneck severity and cycle duration decrease.

#### Single mutants survive if their population size after the first growth phase is larger than the inverse of dilution rate

Once a mutant population is established, it can either disappear because of the bottleneck or survive dilution and pass to the next cycle. For mutants “10,” these two outcomes correspond to two regions of parameter space delimited by line (1)


$$\alpha =\frac{r_{10}-\beta r_{00}}{r_{10}-r_{00}}$$

Indeed, $\alpha >(r_{10}-\beta r_{00})/(r_{10}-r_{00})$ is equivalent to having $Z_{10}^{\left(n\right)}D_{n}\gg 1$ with $Z_{10}^{\left(n\right)}$ the number of “10” mutants after one growth phase (see Supplementary File S1), and in that case the probability that at least one mutant “10” survives dilution goes to 1 as n→+∞. Thus, above line (1), the “10” mutant population survives the bottleneck w.h.p. and because the dilution factor is constant, it will also survive every subsequent bottleneck. The “10” population will then continue growing, allowing double mutants to arise. Below this line, the “10” mutant population at the end of the first cycle is too small to survive dilution, but re-establishes from the WT w.h.p. at each new cycle of the experiment. For “01” mutants, the delimitation between establishment and survival is line (3)


$$\alpha =\frac{\delta r_{01}-\beta r_{00}}{r_{01}-r_{00}}$$

This line is analogous to line (1) except that mutant “01” appears later (when population size reaches $∼n^{\delta }$) but grows faster. Thus lines (1) and (3) delineate four different zones that we named the *Southwest*, *Southeast*, *Northwest* and *Northeast* corners. These zones can be further delimited into different colored areas depending on the value of α (Fig. 2).

#### Southwest corner: no adaptation

In the Southwest corner, no single mutant population can survive. When α<δ (purple area under line (2)), only “10” mutants can establish, they never survive but re-establish w.h.p. at each new cycle. Above line (2), in the green area, both “10” and “01” mutants establish at each cycle but w.h.p. never survive. In these purple and green areas, the probability for single mutants to survive (and thus, for double mutants to establish) goes to 0 as n→+∞ as long as the number of cycles performed is finite. Of course, this is not true anymore for real populations for which n is finite. In that case, we can expect to see weakly beneficial single mutants survive when performing $O(n^{\alpha -\beta -(\alpha -1)r_{10}/r_{00}})$ cycles. When α increases above line (4), the time duration of a growth phase increases and the final population size of “01” mutants becomes large enough for double mutants to arise and establish. These double mutants are lost in dilution w.h.p., except for high values of $r_{11}$ for which they are able to survive even though neither “01” nor “10” survive themselves (case not represented in Fig. 2).

#### Southeast corner: adaptation via “10”

In the Southeast corner, only mutants “10” and “11” survive. Depending on the sign of α−δ, we are either in the blue area with no “01” mutant or in the yellow one with “01” establishing repeatedly but not surviving bottlenecks. The example scenario displayed in Fig. 1d corresponds to the blue area.

#### Northwest corner: adaptation via “01”

In the Northwest corner, only mutants “01” and “11” survive. The “10” mutant population re-establishes at each cycle. If α increases above line (5), the growth phase lasts sufficiently long for the size reached by the “10” mutant population to also produce double mutants.

#### Northeast corner: adaptation via both “10” and “01”

In the Northeast corner (in red), all transitions are observed and all mutants will eventually establish and survive if enough cycles are performed.

### Effect of demography on the timing of establishment of the double mutant

Here we show how demographic parameters affect the timing of adaptation, in particular at which cycle double mutants will establish. In Fig. 2, lines (4) and (5) determine which value of α is needed for double mutants to establish during the first cycle. Above line (4), double mutants arise during the first cycle from mutant “01,” and above line (5) they arise during the first cycle from mutant “10.” Thus, as soon as α is above one of these two lines, double mutants establish during the first cycle. Double mutants may also take more time to establish, as we now explain.

**Fig. 3.**
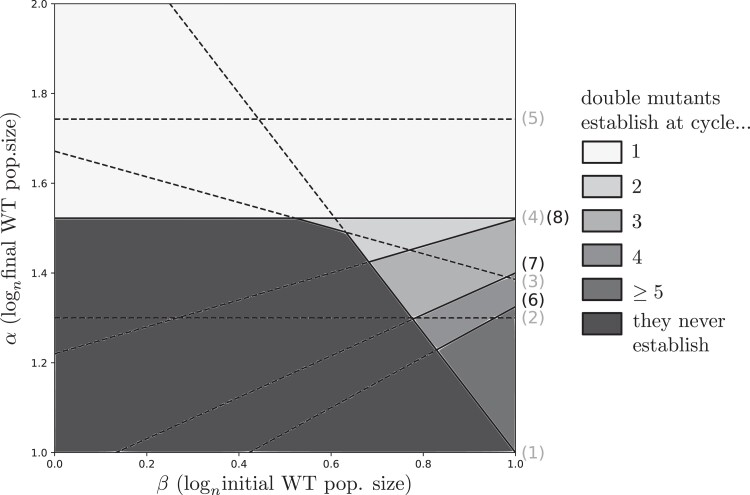
Predicted number of cycles to wait before the establishment of double mutants, as a function of demographic parameters β and α. These predictions are made for infinitely large populations and vanishing mutation rates (δ=1.3, $r_{00}=0.2$, $r_{10}=0.35$, and $r_{01}=0.9$).

The different scenarios of double mutant establishment are illustrated in Fig. 3, using the same parameter values as in Fig. 2. Different shades of gray are used to indicate the speed at which the double mutants are predicted to establish. New lines (6–8) delineate these regions (see equations in Supplementary File S1). See Supplementary Fig. S5a from Supplementary File S1 for the observed number of cycles at which the double mutant establishes in simulations with a finite n. It is clear from Fig. 2 that in the limit where n→+∞, double mutants cannot establish in a finite number of cycles in the bottom-left black zone, and that they establish at the first cycle in the top white zone. In the bottom-right gray zones, double mutants are predicted to establish in a number of cycles which is greater than 1 but w.h.p. finite. Lines (6), (7), and (8) indicate at which cycle single mutant populations are large enough for double mutants to establish. Interestingly, in this area, the value $\beta =\alpha -(\alpha -1)r_{10}/r_{00}$ [corresponding to line (1)] maximizes the speed of double mutants establishment.

### Simulations with density-dependent division rate

In order to test our theoretical predictions in a more realistic setting, we performed simulations with density-dependent growth rates: intrinsic division and death rates are multiplied by $1-(N_{\mathrm{tot}}/K)$, with $N_{\mathrm{tot}}$ the current, total population size, and K the carrying capacity. We consider the case where mutant subpopulations have a small advantage in exploiting the available resources, resulting in a higher carrying capacity. We thus have $K_{\mathrm{WT}}=n^{\alpha }$ for the WT and $K_{\mathrm{mut}}=n^{\alpha (1+s)}$ for mutants. This assumption is necessary to observe the blue bottom-right area from Fig. 2 where the strongly beneficial mutation can only establish in the “10” background. We further assume that dilution arises when the WT population reaches a proportion p of its carrying capacity. More details about our simulations can be found in Supplementary File S1, and Supplementary Fig. S4 provides examples of simulated populations.

**Fig. 4.**
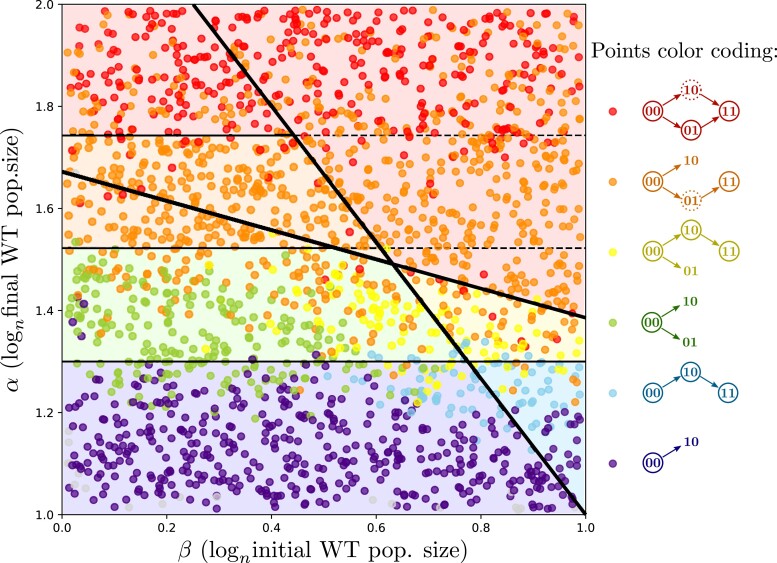
Observed paths in simulations with density-dependent division: each point corresponds to a simulation and is colored according to the observed scenario. Each simulation was run for seven cycles, with parameters $n=10^{12}$, p=0.5, s=0.1, δ=1.3, $r_{00}=0.2$, $r_{10}=0.35$, $r_{01}=0.9$, $r_{11}=1$ and death rates equal to 0.1. Boundaries and background colors are theoretical predictions for exponential growth and large n from Fig. 2.

Figure 4 shows which scenarios were observed after simulating the evolution of a population with 2,000 different pairs of parameters (α,β) and p=0.5. The color of a point corresponds to the evolutionary paths observed during the simulation, with the color coding used in Fig. 2. Here the WT is able to reach only 50% of its carrying capacity before dilution, thus the dynamics are very similar to our first model without density-dependence, until one of the mutants reaches the stationary phase. In Fig. 4, we observe almost all the areas predicted by our model, with blurred boundaries due to stochasticity. However, the scenario where adaptation occurs through both weakly beneficial and strongly beneficial single mutants (red area) is less observed than in density-independent predictions because of competition: when both single mutants survive the first bottleneck, the weakly beneficial mutant “10” is driven to extinction by the strongly beneficial mutant “01” and thus is not able to produce a lot of double mutants (see Supplementary Fig. S4b).

As we discuss in Section 9.2 and Supplementary Fig. S3 of Supplementary File S1, increasing the value of p or the number of cycles increases the probability that some mutants establish and/or survive in areas where they were not expected to, thus shifting the position of certain areas compared to model predictions.

In conclusion, when doing simulations with finite values of n and density-dependent growth, we recover the qualitative effects of α and β on evolutionary pathways. Note, however, that density-dependence reduces the occurrence of the fixation of double mutants via two different pathways (i.e. red dots in Fig. 4).

## Discussion

We characterize the trajectory and the speed of adaptation of an asexual, exponentially growing population subject to periodic bottlenecks. We studied adaptation on a minimal fitness landscape where only two classes of mutations are available: high-rate weakly beneficial mutations and low-rate strongly beneficial mutations.

Our main result is that (1) depending on initial and final population sizes, a unique evolutionary path unfolds and that (2) varying these two parameters, all paths can be explored. Establishment of a mutant is possible when the population size is of the order of the inverse of the relevant mutation rate. Surviving the bottleneck is possible when the final population size is of the order of the bottleneck severity. Tuning initial and final population sizes enables us to determine the evolutionary paths that the population will follow. A particularly interesting implication is that evolutionary paths can appear constrained not only because of sign epistasis (Weinreich *et al.* 2006) and rugged landscapes (Handel and Rozen 2009; de Visser and Krug 2014; Hartl 2014) but also because of fluctuating demography limiting the mutational input and causing the loss of beneficial mutations. This phenomenon had been observed in several previous experimental studies (Garoff *et al.* 2020; Mahrt *et al.* 2021; Schenk *et al.* 2022), and here we dissected the mechanisms by which demographic parameters can constrain and direct mutational pathways.

### Effect of demographic parameters

Our model predicts which evolutionary paths will be observed, in the limit where the population size is large and the mutation rate is small. The predicted outcome depends both on the initial population size (β in logarithmic scale) and the final WT population size (α in logarithmic scale), where 1 in logarithmic scale corresponds to the inverse of the higher mutation rate. As α or β decreases, the accessibility of the double mutant decreases until it is no longer possible for the population to acquire both mutations. Indeed, decreasing population size at the end of the growth phase (decreasing α) limits the supply of mutations and increasing bottleneck severity (decreasing β) prevents mutant populations to survive until the next cycle.

Demographic parameters affect the rate of evolution. Increasing the final population size (α) always speeds up adaptation, as a large population size favors the emergence of mutations and also gives more time for the mutant subpopulation to reach a size large enough to survive the bottleneck. Interestingly, increasing the initial population size (β) has more complex effects. If the final population size is above the threshold for the establishment of the double mutant from the strongly beneficial “01” mutant at the first cycle (α above line (4)), then the initial population size has no influence on the outcome. However, when the final population size is smaller than this threshold, establishment of the double mutant is fastest (in terms of number of cycles) for an intermediate initial population size. Indeed, when β is too small, the bottleneck is too severe for mutations to survive. On the contrary if it is too large, then the bottleneck is less severe but the growth phase is shorter, leading to an overall effect of slowing down double mutant establishment. This non-monotonous effect of β is similar to that of the dilution ratio in Wahl *et al.* (2002): a small ratio allows few mutations to survive, but a high ratio reduces the duration of a cycle and yields fewer mutations.

### Limitations

Our analysis relies on both a large population size and a small mutation rate approximation. However, these approximations correctly predict the outcome even for a finite population size. As shown in Supplementary Fig. S2, boundaries between different scenarios are blurred due to stochasticity but still visible. An important limitation of our model is that the population grows exponentially and is only bounded by the periodic bottlenecks, not by resource limitation. Coupled to the fact that the dilution ratio is kept constant throughout the experiment, this unlimited growth allows different clones to coexist indefinitely without interfering, which does not seem realistic (Miralles *et al.* 1999; Lang *et al.* 2013; Maddamsetti *et al.* 2015). If instead the total inoculum size were constant, we would observe clonal interference (Campos and Wahl 2009, 2010). However, we do observe clonal interference when relaxing the assumption of exponential growth in a set of additional simulations with density-dependence. In this setting, the emergence of double mutants from both single mutants is no longer possible. Nevertheless, these simulations show that the rest of our results hold qualitatively when weakly beneficial mutants can reach a higher population size than WTs. The mutants can reach a higher final population size than WT when the WT does not reach stationary phase at the end of the cycle, or because beneficial mutations can enable a larger stationary size: for example, in the long-term evolution experiment, the evolution of the ability to use citrate causes a 10-fold increase in final optical density, a proxy for population size (Blount *et al.* 2008).

### Application to experimental design

Can our results be used to guide the design of evolution experiments? If mutation and division rates are known, it is possible to choose the size of the inoculum and of the final population size (or carrying capacity, for our model with density-dependence) to decide which mutants emerge and when. For example, if the goal is to obtain double mutants as quickly as possible from 10 mL of a bacterial population saturating at $10^{9}$ individuals/ml with a beneficial mutation rate of $10^{-7}$, then we would recommend based on our model to have a dilution factor of $n^{-(\alpha -1)r_{10}/r_{00}}=(10^{-3}{)}^{r_{10}/r_{00}}$ (corresponding to the optimal value of β mentioned above). More details for computing the optimal dilution factor are available in Supplementary File S1. However all configurations may not be accessible in any given population or species, depending on the mutation rate and distribution of fitness effects. For example, when $r_{10}$ and $r_{01}$ are close to $r_{00}$ (weak selection), the lines (1) and (3) in Fig. 2 are almost vertical. Thus we observe a greater diversity of scenarios when beneficial mutations confer a substantial fitness advantage, i.e. under strong selection.

In a more general setting where we have k beneficial mutations with a similar rate-benefit trade-off, and even without knowing precisely the mutation rate and distribution of fitness effects, what remains true is that increasing the initial and/or final population size will allow the population to access more evolutionary paths. Furthermore, a large initial population size combined with a small final population size (relaxed bottlenecks—short cycles) will favor paths going first through frequent mutations, while a small initial population size with a large final population size (severe bottlenecks—long cycles) will favor paths where the first mutations are rare but strongly beneficial.

The possibility to speed up the rate of adaptation by tuning demographic parameters could also alleviate the problem of bottlenecks in directed evolution (Bloom and Arnold 2009; Badran and Liu 2015).

### Interpretation of experiment outcome

Our results imply that sign epistasis is not necessary for evolution to follow a specific evolutionary path over others. For example, Fig. 1d illustrates a scenario corresponding to parameter values falling into the blue bottom-right area of Fig. 2: the emergence of the strongly beneficial “01” mutant is highly unlikely, but the weakly beneficial “10” mutant establishes in the first cycle and the double mutant establishes during the second cycle. Without prior knowledge on traits and demography, an interpretation of the emergence of “11” mutants exclusively from “10” and never from “01” mutants is sign epistasis: the fitness of the “01” mutant is lower than the WT, but this mutation confers a benefit in the background of the other (weakly beneficial) mutation. However, this interpretation is incorrect here: the strongly beneficial mutation is beneficial in all backgrounds but emerges from “10” and not from “00,” simply because “10” mutants reach a higher population size than the WTs during the course of the experiment. The first set of beneficial mutations could thus enable access to other rarer mutations not through epistatic relationship but a larger final population size.

The phenomenon that we highlight here has been evidenced in experimental evolution. For example, Garoff *et al.* (2020) experimentally evolved *E. coli* under ciprofloxacin antibiotic in bottlenecks of varying severity. They showed that when the final population size is small, evolving mutations are weakly beneficial but affect mechanisms with large mutational target, for example, efflux pump repressors. Rarer and more beneficial mutations only evolve when the final population size is larger. A similar observation has been made by Schenk *et al.* for β-lactam antibiotic resistance (Schenk *et al.* 2022).

All in all, these new mathematical results shed light on the factors shaping adaptation in repeatedly bottlenecked populations, showing that all paths can be followed by adaptation depending on demographic controls, and that the repeated appearance of specific evolutionary paths over others does not imply sign epistasis. This work calls for models studying the effect of demographic controls on evolution in more complex fitness landscapes and for inference methods disentangling the role of epistasis and demography in realized evolution experiments.

## Supplementary Material

iyad001_Supplementary_Data

## Data Availability

Supplementary File S1 contains supplementary information about our model (with derivation of the equations for threshold lines from Figs. 2 and 3) and about stochastic simulations. Code used to perform simulations of the model and generate Fig. 4 can be found at https://github.com/JasmineGamblin/periodic-bottlenecks. Supplemental material available at *GENETICS* online.
