# Supplementary material for: Bottlenecks can constrain and channel evolutionary paths
Source: Genetics. 2023 Feb 2;224(2):iyad001. doi: 10.1093/genetics/iyad001 (PMC10213489; doi:10.1093/genetics/iyad001)
Supplement: iyad001_Supplementary_Data [file iyad001_supplementary_data.pdf]

File S1: Supplementary Information

Bottlenecks can constrain and channel evolutionary paths

Jasmine Gamblin, Sylvain Gandon, François Blanquart and Amaury Lambert

December 23, 2022

In this Supplementary Information we provide more details on our model (section 1), with proofs of results concerning the timing of mutations (sections 2 and 3) and the number of single mutants after one growth phase (sections 4 and 5). These results then allow us to derive the equations for threshold lines from Figure 2 and 3 of the main text (sections 6, 7, and 8). We also describe simulation results for exponential and density-dependent growths (section 9), and explain how to compute the optimal dilution factor (section 10).

Supplementary Text

1 A semi-deterministic model **2**

1.1 A few known results on birth-death processes . . . 2

2 Waiting time to the first mutation **3**

3 Timing of weakly beneficial mutations **4**

4 Number of mutants '10' after one growth phase **4**

4.1 Distribution of Z_{10} . . . 4

5 Number of mutants '01' after one growth phase **5**

5.1 Case $\alpha < \delta$ . . . 5

5.2 Case $\alpha > \delta$ . . . 5

6 Survival of single mutants **5**

7 Constrained evolutionary paths **6**

8 Establishment of double mutants **6**

9 Simulations **7**

9.1 Exponential growth . . . 7

9.2 Density-dependent growth . . . 8

10 Optimal dilution factor **9**

A Appendix: Limit of $Z_{10}^{(n)}$ for $n \to +\infty$ **9**

A.1 Notations . . . 9

A.2 Laplace transform of $Z_{10}^{(n)}/n^q$ . . . 10

Supplementary Figures

S1 Distribution in time of weakly beneficial mutational origins during first cycle 13
S2 Observed paths in simulations with exponential VS density-dependent growth 14
S3 Influence of density-dependence and the number of cycles 15
S4 Examples of simulated trajectories 16
S5 Observed cycles of double mutant emergence in simulations with exponential VS density-dependent growth 17

1 A semi-deterministic model

As described in the main text, we use a semi-deterministic model in order to study the evolution of an asexual population experiencing periodic bottlenecks. The results presented in sections 2 to 5 of this Supplementary Information focus on the first growth phase of this population. During this phase, the wild-type population grows exponentially from a size n^β ($\beta \in (0,1)$) at time 0 to a size n^α ($\alpha > 1$) at time t_n. The wild-type population size at time $t \in [0, t_n]$ is $N_t = N_0 e^{r_{00}t}$. r_{00} is the growth rate and can be decomposed as $r_{00} = b_{00}(1-\mu) - d_{00}$, with b_{00} the division rate, μ the beneficial mutation rate and d_{00} the death rate.

We suppose that two categories of beneficial mutations can arise: weakly beneficial mutations with rate $\mu_{\text{high}} = \frac{1}{n}$ and strongly beneficial mutations with rate $\mu_{\text{low}} = \frac{1}{n^\delta}$ ($\delta > 1$). We focus here on weakly beneficial mutations and thus on mutants with genotype '10' (i.e. having a weakly beneficial mutation but no strongly beneficial mutation). Weakly beneficial mutations arise on the wild-type population according to a time-inhomogeneous Poisson process with parameter $\Lambda(t) = b_{00}\mu_{\text{high}}N_t$. Each one of these mutations gives rise to a clone of mutants '10' following a birth-death process with birth rate $b_{10}(1-\mu_{\text{low}})$ and death rate d_{10}.

1.1 A few known results on birth-death processes

We denote by Y_t the random variable measuring the size of a clone arising from a weakly beneficial mutation after a growth time of t. Then it can be shown (e.g. in [1]) that:

$$\mathbb{P}(Y_t = 0) = \frac{d_{10}(1-e^{-r_{10}t})}{b_{10}(1-\mu_{\text{low}}) - d_{10}e^{-r_{10}t}}$$

$$\mathbb{P}(Y_t = k) = (1-\mathbb{P}(Y_t=0))\left(1 - \frac{b_{10}(1-\mu_{\text{low}})}{d_{10}}\mathbb{P}(Y_t=0)\right)\left(\frac{b_{10}(1-\mu_{\text{low}})}{d_{10}}\mathbb{P}(Y_t=0)\right)^{k-1} \quad \text{for } k>0$$

We denote by $s_{10}(t)$ the probability for this clone to escape stochastic extinction at least until time t. Then:

$$s_{10}(t) = 1 - \mathbb{P}(Y_t=0) = \frac{r_{10}}{b_{10}(1-\mu_{\text{low}}) - d_{10}e^{-r_{10}t}}.$$

For $k > 0$, we can rewrite $\mathbb{P}(Y_t = k)$ as:

$$\mathbb{P}(Y_t = k) = (1-\mathbb{P}(Y_t=0))e^{-r_{10}t}s_{10}(t)\left(1-e^{-r_{10}t}s_{10}(t)\right)^{k-1}.$$

Thus

$$\mathbb{P}\left(Y_t = k | Y_t > 0\right) = e^{-r_{10}t}s_{10}(t)\left(1-e^{-r_{10}t}s_{10}(t)\right)^{k-1}.$$

This shows that Y_t conditioned on survival is a geometric random variable with parameter

$$p_{10}(t) = e^{-r_{10}t}s_{10}(t) = \frac{e^{-r_{10}t}r_{10}}{b_{10}(1-\mu_{\text{low}}) - d_{10}e^{-r_{10}t}}.$$

It follows that the expected clone size at time t conditioned on the clone survival until t is

$$\mathbb{E}\left(Y_t|Y_t > 0\right) = \frac{1}{p_{10}(t)}.$$

We denote this quantity as $c_{10}(t)$ in section 3.

2 Waiting time to the first mutation

We denote by T_n the time at which the first weakly beneficial mutation to escape stochastic extinction occurs. We define σ_n as the time at which the wild-type population reaches a size of n during the first growth phase. As the WT population size has an exponential growth $N_t = N_0 e^{r_{00}t}$ with $N_0 = n^\beta$, we have $\sigma_n = \frac{1-\beta}{r_{00}}\ln(n)$. In this section, we show that $T_n - \sigma_n = O(1)$ for $n \to \infty$. If we denote by U_n the random variable equal to $T_n - \sigma_n$ and take $t > -\sigma_n$, then

$$\mathbb{P}(U_n \le t) = \mathbb{P}\left(T_n \le t + \sigma_n\right).$$

Weakly beneficial mutations arise according to a non-homogeneous Poisson process of parameter $\Lambda(t) = b_{00}\mu_{\text{high}} n^\beta e^{r_{00}t}$, and the probability that a mutant '10' escapes stochastic extinction is $s_{10}(\infty) = \frac{r_{10}}{b_{10}(1-\mu_{\text{low}})}$. Thus the probability that no such mutant occurs before time $t + \sigma_n$ is

$$\mathbb{P}\left(T_n > t + \sigma_n\right) = \exp\left(-\int_0^{t+\sigma_n} \Lambda(s)\frac{r_{10}}{b_{10}(1-\mu_{\text{low}})}\,\mathrm{d}s\right).$$

Thus

$$\begin{aligned}
\mathbb{P}(U_n \le t) &= 1 - \exp\left(-\int_0^{t+\frac{1-\beta}{r_{00}}\ln(n)} b_{00}\mu_{\text{high}} n^\beta e^{r_{00}s}\frac{r_{10}}{b_{10}(1-\mu_{\text{low}})}\,\mathrm{d}s\right) \\
&= 1 - \exp\left(\frac{b_{00}\mu_{\text{high}} r_{10}}{b_{10}(1-\mu_{\text{low}})r_{00}} n^\beta \left(1 - e^{r_{00}t + \ln(n)(1-\beta)}\right)\right) \\
&= 1 - \exp\left(\frac{b_{00}\mu_{\text{high}} r_{10}}{b_{10}(1-\mu_{\text{low}})r_{00}} n^\beta\right)\exp\left(-\frac{b_{00}\mu_{\text{high}} r_{10}}{b_{10}(1-\mu_{\text{low}})r_{00}} n e^{r_{00}t}\right).
\end{aligned}$$

Recalling that $\mu_{\text{high}} = \frac{1}{n}$ and $\mu_{\text{low}} = \frac{1}{n^\delta}$ with $\delta > 1$, we obtain

$$\mathbb{P}(U_n \le t) \underset{n\to\infty}{\to} 1 - \exp\left(-\frac{b_{00}r_{10}}{b_{10}r_{00}}e^{r_{00}t}\right).$$

The limit of the cumulative distribution function of U_n shows that it converges in distribution to a random variable U_∞, where $-U_\infty$ follows a Gumbel distribution with parameters $\left(\frac{1}{r_{00}}\ln\left(\frac{b_{00}r_{10}}{b_{10}r_{00}}\right), \frac{1}{r_{00}}\right)$. Thus we have:

$$T_n - \sigma_n \underset{n\to\infty}{=} O(1).$$

This shows that the first weakly mutation to escape stochastic extinction occurs around the time where the WT population size is of the order of the inverse of the mutation rate.

3 Timing of weakly beneficial mutations

Here we look more closely at the times when weakly beneficial mutations occur during the first growth phase. We are interested in mutant clones that survive the first bottleneck, and we seek to investigate how their mutational origins are distributed over time. Mutational events giving birth to a surviving clone are called **surviving** mutations (see main text). The rate at which these mutations are produced at time t during the first growth phase is

$$b_{00}\mu_{\text{high}}N_t \propto e^{r_{00}t}.$$

Thus most mutations arrive at the end of the growth phase. Their distribution is plotted in pink on Figure S1. However, early mutations have more descendants. The final expected clone size (conditioned on survival) for a weakly beneficial mutation occurring at time t is $c_{10}(t) = \frac{1}{r_{10}}\left(b_{10}(1-\mu_{\text{low}})e^{r_{10}(t_n-t)} - d_{10}\right)$. The probability that a mutation occurring at time t survives until the end of the growth phase being $s_{10}(t) = \frac{r_{10}}{b_{10}(1-\mu_{\text{low}})-d_{10}e^{-r_{10}(t_n-t)}}$, the rate of weakly beneficial mutations weighted by final expected clone size at time t is

$$b_{00}\mu_{\text{high}}N_t s_{10}(t)c_{10}(t) \propto e^{-(r_{10}-r_{00})t}$$

(see section 1 for a derivation of c_{10} and s_{10}). This distribution is plotted in purple on Figure S1. The consequence is that clones that manage to survive the bottleneck have more chance to arise from a mutation arising in the middle of the growth phase (see solid blue curve on Figure S1). Indeed, if we suppose that the dilution is a binomial sampling, then the rate of surviving weakly beneficial mutations at time t is:

$$b_{00}\mu_{\text{high}}N_t s_{10}(t)\left(1-\left(1-n^{-(\alpha-\beta)}\right)^{c_{10}(t)}\right)$$

This distribution was already studied by Wahl et al. in a setting where the selective advantage is small [2]. They found that surviving mutations were likely to occur at all times during a growth phase, with a relatively flat distribution. This is indeed what we can observe on Figure S1 when $r_{10} \simeq r_{00}$ (dashed blue curve).

4 Number of mutants '10' after one growth phase

$Z_{10}^{(n)}$ is the random variable counting the number of mutants with genotype '10' at the end of the first growth phase. In the appendix, we show that $Z_{10}^{(n)} \underset{n\to+\infty}{\sim} n^{(\alpha-1)\frac{r_{10}}{r_{00}}} Z_{10}$. This shows in particular that mutants '10' **emerge** during the first growth phase, ie. they reach a large population size ($Z_{10}^{(n)} \gg 1$).

4.1 Distribution of Z_{10}

The characteristic function of Z_{10} is

$$\begin{aligned}
\phi_{Z_{10}}(t) &= \mathcal{L}_{Z_{10}}(-it) \\
&= \exp\left(-\frac{b_{00}\pi}{b_{10}\sin\left(\frac{r_{00}\pi}{r_{10}}\right)}\left(\frac{b_{10}}{r_{10}}\right)^{\frac{r_{00}}{r_{10}}} |t|^{\frac{r_{00}}{r_{10}}}\left(-i\,\text{sgn}(t)\right)^{\frac{r_{00}}{r_{10}}}\right) \\
&= \exp\left(-\frac{b_{00}\pi}{b_{10}\sin\left(\frac{r_{00}\pi}{r_{10}}\right)}\left(\frac{b_{10}}{r_{10}}\right)^{\frac{r_{00}}{r_{10}}} \cos\left(\frac{\pi r_{00}}{2r_{10}}\right)|t|^{\frac{r_{00}}{r_{10}}}\left(1-i\,\text{sgn}(t)\tan\left(\frac{\pi r_{00}}{2r_{10}}\right)\right)\right).
\end{aligned}$$

This expression tells us that Z_{10} has a one-sided (Lévy) stable distribution supported by $[0, \infty)$ [3]. The stability parameter is $\frac{r_{00}}{r_{10}} \in (0,1)$, which implies that all moments are infinite. Sadly, the probability density function of Z_{10} is not analytically expressible in the general case [4], nor are its median and mode.

5 Number of mutants '01' after one growth phase

For mutants with genotype 01, there are two different cases depending on the sign of $\alpha - \delta$. Indeed, the probability that no mutant '01' appears during a growth phase is:

$$\begin{aligned} p_{01}^{(n)} &= \exp\left(-\int_0^{t_n} b_{00}\mu_{\text{low}} N_t \,\mathrm{d}t\right) \\ &= \exp\left(-b_{00} n^{\beta-\delta} \int_0^{t_n} e^{r_{00}t}\,\mathrm{d}t\right) \\ &= \exp\left(-b_{00} n^{\beta-\delta} \frac{1}{r_{00}} (n^{\alpha-\beta} - 1)\right) \\ &= \exp\left(-\frac{b_{00}}{r_{00}} (n^{\alpha-\delta} - n^{\beta-\delta})\right) \end{aligned}$$

5.1 Case $\alpha < \delta$

In this case, $p_{01}^{(n)}$ goes to 1 as $n \to \infty$. It is highly unlikely to see any '01' mutant appear during the course of the experiment.

5.2 Case $\alpha > \delta$

In that case, $p_{01}^{(n)}$ goes to 0 as $n \to \infty$. Furthermore, we can do the same computations as with mutants '10' and find that the random variable $Z_{01}^{(n)}$ counting the number of mutants '01' at the end of the first growth phase verifies:

$$Z_{01}^{(n)} \underset{n\to+\infty}{\sim} n^{(\alpha-\delta)\frac{r_{01}}{r_{00}}} Z_{01}$$

with Z_{01} a stable distribution with stability parameter $\frac{r_{00}}{r_{01}}$. This shows that mutants '01' emerge during the first growth phase.

6 Survival of single mutants

From Section 4 we know that mutants '10' **establish** with high probability during the first cycle. However this does not guarantee that they will **survive**, that is, reach a sufficiently large size to survive the next bottleneck. Mutants '10' survive if $A = -(\alpha - \beta) + (\alpha - 1)\frac{r_{10}}{r_{00}}$ is positive. Indeed the expected quantity of mutants '10' present at the beginning of the second cycle is $D_n Z_{10}^{(n)}$, with $D_n = n^{-(\alpha-\beta)}$ being the dilution factor. This quantity is equivalent to $n^{-(\alpha-\beta)+(\alpha-1)\frac{r_{10}}{r_{00}}} Z_{10}$ when n is large, thus the population size of '10' mutants starting in the second cycle is large if $A > 0$ and

negligible if $A < 0$. If $A > 0$, the extinction probability of '10' mutants is close to zero and their growth is deterministic for the remainder of the experiment, so that surviving one bottleneck entails the survival of all bottlenecks. If $A < 0$, with high probability no '10' mutant survives the dilution as $n \to \infty$. Indeed, the probability that no mutant survives dilution knowing that $Z_{10} = z$ is

$$\left(1 - n^{-(\alpha-\beta)}\right)^{zn^{(\alpha-1)\frac{r_{10}}{r_{00}}}} \underset{n\to\infty}{\sim} \exp\left(-zn^{A}\right).$$

Then we discuss the **establishment** and **survival** of the strongly beneficial '01' mutant in the population. It establishes during the first cycle if $\alpha > \delta$. In this case, the number of '01' mutants at the end of the first cycle $Z_{01}^{(n)}$ is equivalent to $n^{(\alpha-\delta)\frac{r_{01}}{r_{00}}} Z_{01}$ for $n \to \infty$. Similarly to '10' mutants, they survive if $-(\alpha-\beta) + (\alpha-\delta)\frac{r_{01}}{r_{00}}$ is positive.

On the contrary if $\alpha < \delta$, then the probability that no '01' mutant is present at time t_n $p_{01}^{(n)}$ goes to 1 as $n \to \infty$ (see section 5). As the WT population has the same growth trajectory in every phase, $p_{01}^{(n)}$ is in fact the probability to have no '01' mutant at the end of any cycle.

7 Constrained evolutionary paths

Parameters α, β and r_{00} govern the demography of the wild-type population, while parameters δ, r_{10} and r_{01} drive the appearance and growth rates of mutants. We have seen in section 6 that their relative values determine which mutations can survive, and a consequence is that they also determine which evolutionary paths are accessible. This is illustrated in Figure 2 of main text, where the different areas are delimited by the following threshold lines:

- (1) $\alpha > \frac{r_{10}-\beta r_{00}}{r_{10}-r_{00}}$ condition for the survival of mutants 10 (this is equivalent to $A > 0$);
- (2) $\alpha > \delta$ condition for the establishment of mutants '01' during first cycle;
- (3) $\alpha > \frac{\delta r_{01}-r_{00}}{r_{01}-r_{00}}$ condition for the survival of mutants 01;
- (4) $(\alpha-\delta)\frac{r_{01}}{r_{00}} > 1$ condition for the establishment of double mutants from mutant '01' during first cycle;
- (5) $(\alpha-1)\frac{r_{10}}{r_{00}} > \delta$ condition for the establishment of double mutants from mutant '10' during first cycle.

Conditions (4) and (5) are explained in the following section.

8 Establishment of double mutants

Demographic parameters constrain evolutionary paths, but also the timing of evolution. In particular, they determine in which cycle the double mutants '11' will establish. In the case where mutants '10' survive the first bottleneck, we can consider that their growth is deterministic during the following cycles. At the end of the kth cycle they have gone through $k-1$ dilutions and deterministic growth phases, thus their population size is

$$n^{(\alpha-1)\frac{r_{10}}{r_{00}}} Z_{10} * D_n^{k-1} * (e^{r_{10}t_n})^{k-1}$$
$$\underset{n\to\infty}{=} O\left(n^{(\alpha-1)\frac{r_{10}}{r_{00}}+(k-1)\left(\frac{r_{10}}{r_{00}}-1\right)(\alpha-\beta)}\right)$$

Thus double mutants produced from mutant '10' establish during the first cycle k_1 such that

$$(\alpha-1)\frac{r_{10}}{r_{00}}+(k_1-1)\left(\frac{r_{10}}{r_{00}}-1\right)(\alpha-\beta)>\delta.$$

Similarly, double mutants produced from mutant '01' establish during the first cycle k_2 such that

$$(\alpha-\delta)\frac{r_{01}}{r_{00}}+(k_2-1)\left(\frac{r_{01}}{r_{00}}-1\right)(\alpha-\beta)>1.$$

As a result, double mutants establish at cycle $\min(k_1,k_2)$. This is illustrated in Figure 3 from main text, where additional lines are plotted compared to Figure 2:

- (6) $(\alpha-1)\frac{r_{10}}{r_{00}}+\left(\frac{r_{10}}{r_{00}}-1\right)(\alpha-\beta)>\delta$ condition for the establishment of double mutants from mutant '10' during the second cycle;
- (7) $(\alpha-1)\frac{r_{10}}{r_{00}}+2\left(\frac{r_{10}}{r_{00}}-1\right)(\alpha-\beta)>\delta$ condition for the establishment of double mutants from mutant '10' during cycle 3;
- (8) $(\alpha-1)\frac{r_{10}}{r_{00}}+3\left(\frac{r_{10}}{r_{00}}-1\right)(\alpha-\beta)>\delta$ condition for the establishment of double mutants from mutant '10' during cycle 4.

Lines representing conditions for the establishment of double mutants from mutant '01' during cycles 2, 3 and 4 are not plotted because they are below lines (6-8) and thus are not relevant for area delimitation.

9 Simulations

The code that was used to run the following simulations is available on Github at `https://github.com/JasmineGamblin/periodic-bottlenecks`, in the form of a Python script and a Jupyter notebook from which simulations can easily be run.

9.1 Exponential growth

We simulated populations evolving according to a fully stochastic model: individuals were grouped by subpopulations carrying the same genotype, each following a birth-death-mutation process. Double mutants originating from the weakly beneficial single mutant or the strongly beneficial single mutant were grouped separately. We used the principle of Gillespie's algorithm [5] to simulate the evolution of the system during growth phases, and binomial sampling to perform the dilution step. We resorted to a τ-leaping approximation to speed-up the simulation when population sizes become large [6].

For each simulation, we recorded at the end of each growth phase which mutant subpopulations were present above a fixed threshold of 50 individuals (chosen so that the probability of extinction from this size is $< 1\%$). This way we could associate a color to the simulation according to the observed evolutionary paths, following the color coding from Figure 2 of main text.

We obtained Figures S2(a,b,c) by randomly drawing 2,000 pairs of parameters (α,β) and simulating a population for 7 cycles, for different values of n. Populations for $n \geq 10^9$ and $\alpha > 1.4$ where simulated

only for 4 cycles to avoid dealing with too large numbers. We can observe that for increasing values of n, the results of the simulations converge to the deterministic behavior that we predicted for $n \to \infty$. Grey points correspond to simulations where no mutant subpopulation was detected above the threshold.

Figure S5(a) was obtained by drawing 2,000 pairs of demographic parameters. For each pair, a population was simulated for 7 cycles and the cycle at which the double mutant emerges was recorded.

9.2 Density-dependent growth

For simulations with density-dependent growth rates, we multiplied intrinsic birth and death rates by $1 - \frac{N_{tot}}{K}$, with N_{tot} the total population size and K the carrying capacity. We chose $K_{\text{WT}} = n^{\alpha}$ for wild-types and $K_{\text{mut}} = n^{\alpha(1+s)}$ for all mutant subpopulations, with s being a small advantage that mutants may have in exploiting the available resources. We also introduced a parameter p which is the proportion of the carrying capacity that wild-types are able to reach before dilution. Hence, we now have

$$t_n = \frac{1}{r_{00}} \ln\left(\frac{n^{\alpha-\beta} - 1}{\frac{1}{p} - 1}\right)$$

and

$$D_n = \frac{n^{\beta-\alpha}}{p}.$$

Figure S4 shows examples of simulated evolution for 11 different combinations of parameters α and β. We chose parameter values $p = 0.5$ and $s = 0.1$, thus wild-types are able to reach 50% of their carrying capacity before dilution. Here the fact that growth is density-dependent introduces competition between the different subpopulations: we observe that wild-types and small-benefit mutants can be driven to extinction by fitter mutants. This effect of competition can be observed on Figures S2(d,e,f), where we see fewer red points in comparison to simulations with exponential growth (Figures S2(a,b,c)). This is due to the fact that in most cases the weakly beneficial single mutant is outcompeted by the strongly beneficial one and thus is not able to produce double mutants.

Figure S3 shows the effect of two parameters: the proportion p of the carrying capacity reached by wild-types at the end of the growth phase, and the number of cycles performed. When varying p between 0.5 and 0.9, we can verify that the case $p = 0.5$ is very similar to the exponential case as we would expect (because at this point the growth is still nearly exponential). For a higher value of p, we observe that strongly beneficial single mutants have some chance so establish even if $\alpha < \delta$, and that both single mutants may survive dilution even below their survival thresholds. These observations are due to the fact that with density-dependent growth it takes more time for wild-types to reach their final population size compared to the exponential case. Thus the cycle duration is longer, giving more time to mutants to establish and/or survive. When varying the number of cycles performed between 7 and 30, we observe as expected that performing more cycles also increases the probability that mutants establish and/or survive in areas where they are not expected to do so in large n predictions.

Figure S5(b) was obtained by drawing 2,000 pairs of demographic parameters. For each pair, a population was simulated for 7 cycles and the cycle at which the double mutant emerges was recorded.

10 Optimal dilution factor

If for a given population we know the maximum density m, the volume v and the beneficial mutation rate μ, then we can compute the parameters of our model:

$$n = \mu^{-1} \quad \text{and} \quad \alpha = \frac{\ln(mv)}{\ln(n)}.$$

For a rare, strongly beneficial mutation with rate μ_{low}, we predict that:

- If $\delta = -\frac{\ln(\mu_{\text{low}})}{\ln(n)} < \alpha$, this mutation will establish at the first cycle of growth.
- If not, it may still establish in the background of more frequent but less advantageous mutations if they are able to reach a higher population size than the wild-type. In that case, we can maximize the speed of adaptation by choosing the parameter β to be just above line (1) of Figure 2 from the main text. To be on that line, β must satisfy the equation $\alpha = (r_{10} - \beta r_{00})/(r_{10} - r_{00})$, which leads to an optimal dilution factor of:

$$n^{-(\alpha-1)r_{10}/r_{00}} = (\mu^{-\frac{\ln(mv)}{\ln(\mu)}-1})^{r_{10}/r_{00}}.$$

This optimal dilution factor can be bounded above by $\mu^{-\frac{\ln(mv)}{\ln(\mu)}-1}$ if the growth rates are not known.

A Appendix: Limit of $Z_{10}^{(n)}$ for $n \to +\infty$

Here we show that $Z_{10}^{(n)} \underset{n\to+\infty}{\sim} n^{(\alpha-1)\frac{r_{10}}{r_{00}}} Z_{10}$. We will first introduce some notations, then compute the Laplace transform of $\frac{Z_{10}^{(n)}}{n^q}$ and show that it converges to a non-trivial function for $q = (\alpha - 1)\frac{r_{10}}{r_{00}}$ when $n \to \infty$.

A.1 Notations

We define the following random variables:

$M_{t_n} \in \mathbb{N}$ counts the number of weakly beneficial mutations arising among the WT population and still present at time t_n. M_{t_n} is distributed as a Poisson random variable of parameter $\Lambda(t_n) = \int_0^{t_n} b_{00}\mu_{\text{high}} N_t s_{10}(t_n - t)\,\mathrm{d}t$.

$T_1, ..., T_{M_{t_n}} \in [0, t_n]$ are the times when these mutations arise. Conditioned on $\{M_{t_n} = m\}$, they are independent and identically distributed with probability density $\frac{b_{00}\mu_{\text{high}} N_t s_{10}(t_n - t)}{\Lambda(t_n)}$.

$Y_t^{(i)} \in \mathbb{N}$ is the size after a growth time of t of the clone arising from the ith weakly beneficial mutation. Conditioned on $\{M_{t_n} = m\}$, the $(Y_t^{(i)})$ with $i \in [1..M_{t_n}]$ are independent and identically distributed. $Y_t^{(i)}$ is a birth-death process starting at time T_i and killed at time t_n, conditioned to survive until t_n. We recalled in section 1 that $Y_t^{(i)}$ is a geometric variable with parameter $p_{10}(t) = \frac{e^{-r_{10}t} r_{10}}{b_{10}(1-\mu_{\text{low}}) - d_{10}e^{-r_{10}t}}$.

With that we can express $Z_{10}^{(n)}$ as:

$$Z_{10}^{(n)} = \sum_{i=1}^{M_{t_n}} Y_{t_n - T_i}^{(i)}$$

A.2 Laplace transform of $Z_{10}^{(n)}/n^q$

Let us compute the Laplace transform of $\frac{Z_{10}^{(n)}}{n^q}$. For $u > 0$, we have:

$$\begin{aligned}
\mathcal{L}_{Z_{10}^{(n)}/n^q}(u) &= \mathbb{E}\left(e^{-u\frac{Z_{10}^{(n)}}{n^q}}\right) \\
&= \mathbb{E}\left(e^{-\frac{u}{n^q}\sum_{i=1}^{M_{t_n}} Y_{t_n-T_i}^{(i)}}\right) \\
&= \mathbb{E}_{M_{t_n}}\left(\mathbb{E}_{T_i|M_{t_n}}\left(\mathbb{E}_{Y_{t_n-T_i}^{(i)}|T_i}\left(e^{-\frac{u}{n^q}Y_{t_n-T_i}^{(i)}}\right)\right)^{M_{t_n}}\right).
\end{aligned}$$

The Laplace transform of a geometric random variable Y of parameter p is $\mathbb{E}\left(e^{-uY}\right) = \frac{p}{e^u-(1-p)}$, thus

$$\begin{aligned}
\mathcal{L}_{Z_{10}^{(n)}/n^q}(u) &= \mathbb{E}_{M_{t_n}}\left(\mathbb{E}_{T_i|M_{t_n}}\left(\frac{p_{10}(t_n-T_i)}{e^{\frac{u}{n^q}}-1+p_{10}(t_n-T_i)}\right)^{M_{t_n}}\right) \\
&= \mathbb{E}_{M_{t_n}}\left(\left(\int_0^{t_n}\frac{p_{10}(t_n-t)}{e^{\frac{u}{n^q}}-1+p_{10}(t_n-t)} * \frac{b_{00}\mu_{\text{high}}N_t s_{10}(t_n-t)}{\Lambda(t_n)}\,\mathrm{d}t\right)^{M_{t_n}}\right) \\
&= \sum_{m=0}^{\infty}\left(\int_0^{t_n}\frac{p_{10}(t_n-t)}{e^{\frac{u}{n^q}}-1+p_{10}(t_n-t)} * \frac{b_{00}\mu_{\text{high}}N_t s_{10}(t_n-t)}{\Lambda(t_n)}\,\mathrm{d}t\right)^m \frac{\Lambda(t_n)^m}{m!}e^{-\Lambda(t_n)} \\
&= e^{-\Lambda(t_n)}\sum_{m=0}^{\infty}\left(\int_0^{t_n}\frac{p_{10}(t_n-t)}{e^{\frac{u}{n^q}}-1+p_{10}(t_n-t)}b_{00}\mu_{\text{high}}N_t s_{10}(t_n-t)\,\mathrm{d}t\right)^m\frac{1}{m!} \\
&= \exp\left(-\Lambda(t_n)\right)\exp\left(\int_0^{t_n} b_{00}\mu_{\text{high}}N_t s_{10}(t_n-t)\frac{p_{10}(t_n-t)}{e^{\frac{u}{n^q}}-1+p_{10}(t_n-t)}\,\mathrm{d}t\right).
\end{aligned}$$

Then recalling that $\Lambda(t_n) = \int_0^{t_n} b_{00}\mu_{\text{high}}N_t s_{10}(t_n-t)\,\mathrm{d}t$, we have

$$\mathcal{L}_{Z_{10}^{(n)}/n^q}(u) = \exp\left(\int_0^{t_n} b_{00}\mu_{\text{high}}N_t s_{10}(t_n-t)\left(\frac{p_{10}(t_n-t)}{e^{\frac{u}{n^q}}-1+p_{10}(t_n-t)}-1\right)\mathrm{d}t\right)$$

and by replacing $p_{10}(t_n-t)$ and $s_{10}(t_n-t)$ by their expression we obtain:

$$\mathcal{L}_{Z_{10}^{(n)}/n^q}(u) = \exp\left(\int_0^{t_n} b_{00}n^{\beta-1}e^{r_{00}t}r_{10}\frac{1-e^{\frac{u}{n^q}}}{e^{r_{10}(t-t_n)}(b_{10}(1-\mu_{\text{low}})-d_{10}e^{\frac{u}{n^q}})-b_{10}(1-\mu_{\text{low}})(1-e^{\frac{u}{n^q}})}\,\mathrm{d}t\right).$$

Using the change of variables $y = \frac{e^{r_{00}t}-1}{e^{r_{00}t_n}-1}$, $\mathrm{d}y = \frac{r_{00}e^{r_{00}t}}{e^{r_{00}t_n}-1}\,\mathrm{d}t$ leads to:

$$\begin{aligned}
\mathcal{L}_{Z_{10}^{(n)}/n^q}(u) &= \exp\left(-\frac{b_{00}r_{10}}{r_{00}}n^{\beta-1}(n^{\alpha-\beta}-1)\frac{(e^{\frac{u}{n^q}}-1)}{b_{10}(1-\mu_{\text{low}})-d_{10}e^{\frac{u}{n^q}}}\int_0^1\frac{\mathrm{d}y}{\frac{((n^{\alpha-\beta}-1)y+1)^{\frac{r_{10}}{r_{00}}}}{n^{\frac{r_{10}}{r_{00}}(\alpha-\beta)}}+\frac{b_{10}(1-\mu_{\text{low}})(e^{\frac{u}{n^q}}-1)}{b_{10}(1-\mu_{\text{low}})-d_{10}e^{\frac{u}{n^q}}}}\right) \\
&= \exp(g(n)I_n)
\end{aligned}$$

with

$$g(n) = -\frac{b_{00}r_{10}}{r_{00}} n^{\beta-1}(n^{\alpha-\beta}-1)\frac{(e^{\frac{u}{n^q}}-1)}{b_{10}(1-\mu_{\mathrm{low}})-d_{10}e^{\frac{u}{n^q}}}$$

$$\underset{n\to+\infty}{\sim} \frac{-ub_{00}}{r_{00}} n^{\alpha-1-q}$$

and

$$I_n = \int_0^1 \frac{\mathrm{d}y}{\frac{((n^{\alpha-\beta}-1)y+1)^{\frac{r_{10}}{r_{00}}}}{n^{\frac{r_{10}}{r_{00}}(\alpha-\beta)}} + \frac{b_{10}(1-\mu_{\mathrm{low}})(e^{\frac{u}{n^q}}-1)}{b_{10}(1-\mu_{\mathrm{low}})-d_{10}e^{\frac{u}{n^q}}}}.$$

For $n \to \infty$, I_n is equivalent to

$$I_n^* = \int_0^1 \frac{\mathrm{d}y}{y^c + a_n} = \int_0^\infty \frac{e^{-x}}{e^{-cx}+a_n}\mathrm{d}x$$

where $c = \frac{r_{10}}{r_{00}}$ and $a_n = \frac{b_{10}(1-\mu_{\mathrm{low}})u}{r_{10}n^q}$. We can compute this integral using the residue theorem [7]: we integrate the function $h(x) = \frac{e^{-x}}{e^{-cx}+a_n}$ on a rectangle with width R and height $\frac{2\pi}{c}$ with bottom left corner on the origin. If we respectively name I_R, K_R, J_R and L_R the integrals along the four sides of the rectangle, then we have $I_R \underset{R\to\infty}{\to} I_n^*$, $J_R = -e^{-\frac{2i\pi}{c}} I_R$ and $J_R + K_R \underset{R\to\infty}{\to} 0$. As this rectangle contains one simple pole of $h(x)$ (for $x = -\frac{\ln(a_n)}{c} + \frac{i\pi}{c}$), we obtain:

$$I_n^*(1-e^{-\frac{2i\pi}{c}}) = 2i\pi \mathrm{Res}\left(\frac{e^{-x}}{e^{-cx}+a_n}, -\frac{\ln(a_n)}{c} + \frac{i\pi}{c}\right)$$

$$I_n^* = \frac{2i\pi}{1-e^{-\frac{2i\pi}{c}}} * \frac{e^{\frac{\ln(a_n)}{c}-\frac{i\pi}{c}}}{-ce^{-c(-\frac{\ln(a_n)}{c}+\frac{i\pi}{c})}}$$

$$I_n^* = \frac{2i\pi}{e^{\frac{i\pi}{c}} - e^{-\frac{i\pi}{c}}} * \frac{a_n^{\frac{1}{c}}}{ca_n}$$

$$I_n^* = \frac{\pi}{c\sin(\frac{\pi}{c})} a_n^{\frac{1}{c}-1}$$

It follows that

$$I_n \underset{n\to+\infty}{\sim} \frac{r_{00}\pi}{r_{10}\sin\left(\frac{r_{00}\pi}{r_{10}}\right)}\left(\frac{b_{10}u}{r_{10}n^q}\right)^{\frac{r_{00}}{r_{10}}-1}$$

and

$$g(n)I_n \underset{n\to\infty}{\sim} \frac{-b_{00}\pi}{b_{10}\sin\left(\frac{r_{00}\pi}{r_{10}}\right)}\left(\frac{b_{10}u}{r_{10}}\right)^{\frac{r_{00}}{r_{10}}} n^{\alpha-1-\frac{r_{00}}{r_{10}}q}.$$

Thus $\mathcal{L}_{Z_{10}^{(n)}/n^q}(u)$ has a finite limit when $n \to \infty$ for $q = (\alpha-1)\frac{r_{10}}{r_{00}}$:

$$\mathcal{L}_{Z_{10}^{(n)}/n^{(\alpha-1)\frac{r_{10}}{r_{00}}}}(u) \underset{n\to\infty}{\to} \exp\left(-\frac{b_{00}\pi}{b_{10}\sin\left(\frac{r_{00}\pi}{r_{10}}\right)}\left(\frac{b_{10}u}{r_{10}}\right)^{\frac{r_{00}}{r_{10}}}\right).$$

It follows that

$$Z_{10}^{(n)} \underset{n\to+\infty}{\sim} n^{(\alpha-1)\frac{r_{10}}{r_{00}}} Z_{10}$$

with Z_{10} a random variable independent of n with Laplace transform:

$$\mathcal{L}_{Z_{10}}(u) = \exp\left(-\frac{b_{00}\pi}{b_{10}\sin\left(\frac{r_{00}\pi}{r_{10}}\right)}\left(\frac{b_{10}u}{r_{10}}\right)^{\frac{r_{00}}{r_{10}}}\right).$$

References

[1] David G. Kendall. On the generalized "birth-and-death" process. *Annals of Mathematical Statistics*, 19(1):1–15, mar 1948.

[2] Lindi M. Wahl, Philip J. Gerrish, and Ivan Saika-Voivod. Evaluating the impact of population bottlenecks in experimental evolution. *Genetics*, 162(2):961–971, oct 2002.

[3] Jean Bertoin. *Lévy processes*. Cambridge university press, Cambridge, 1996.

[4] Karol A. Penson, K. Górska, and Katarzyna Górska. Exact and explicit probability densities for one-sided Lévy stable distributions. *Physical Review Letters*, 105(21), nov 2010.

[5] Daniel T. Gillespie. Exact stochastic simulation of coupled chemical reactions. *The Journal of Physical Chemistry*, 81(25):2340–2361, dec 1977.

[6] Daniel T. Gillespie. Approximate accelerated stochastic simulation of chemically reacting systems. *Journal of Chemical Physics*, 115(4):1716–1733, jul 2001.

[7] Lars Ahlfors. *Complex Analysis*. McGraw-Hill Inc., New York, 1979.

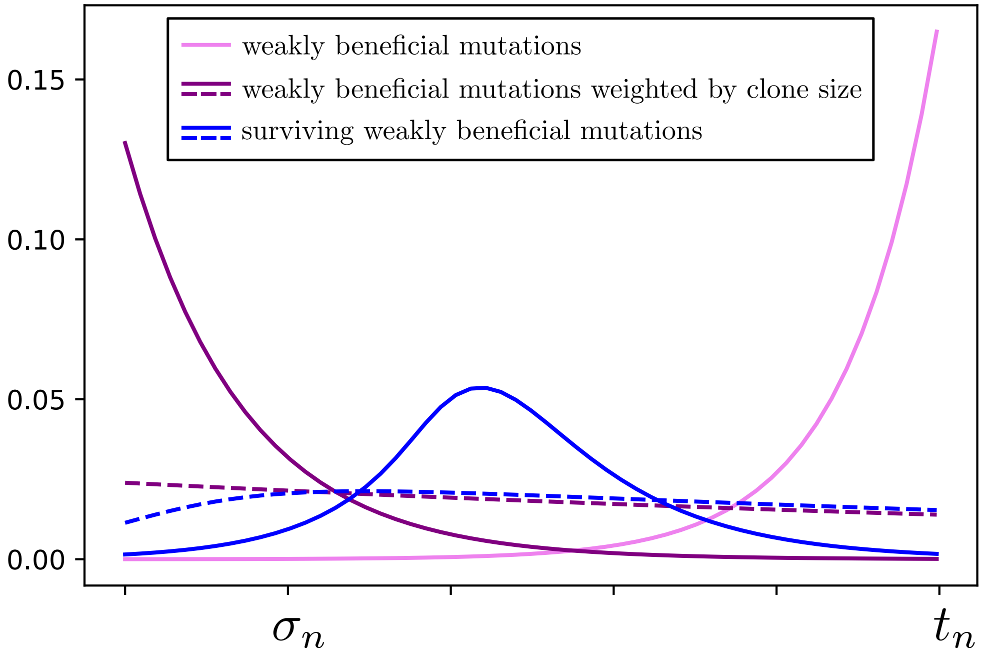

Figure S1: Distribution in time of weakly beneficial mutational origins during first cycle. Pink curve: total weakly beneficial mutation rate, purple curves: total weakly beneficial mutation rate weighted by final expected clone size, and blue curves: rate of emergence of weakly beneficial mutations that survive the first bottleneck. Solid curves are plotted for $r_{10} = 0.3$, dashed ones for $r_{10} = 0.18$ ($r_{00} = 0.17$, $n = 10^8$, $\alpha = 1.4$, $\beta = 0.9$).

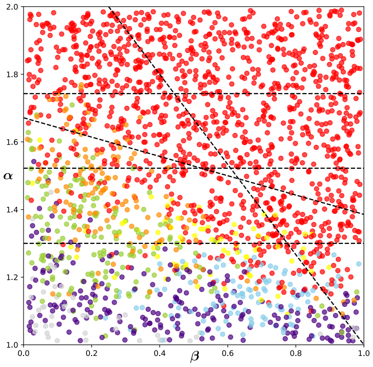

(a) Exponential growth, $n = 10^6$

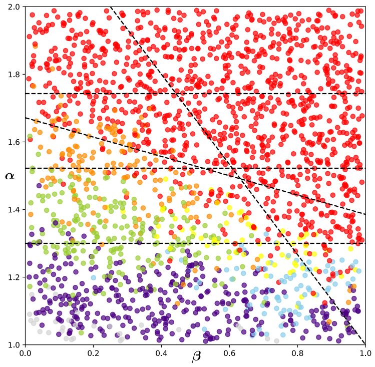

(b) Exponential growth, $n = 10^9$

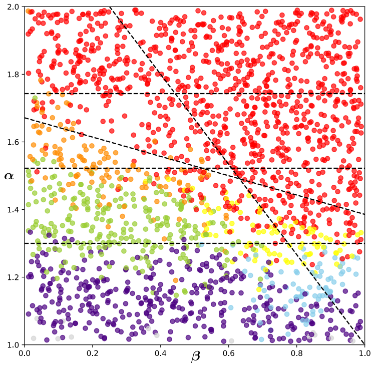

(c) Exponential growth, $n = 10^{12}$

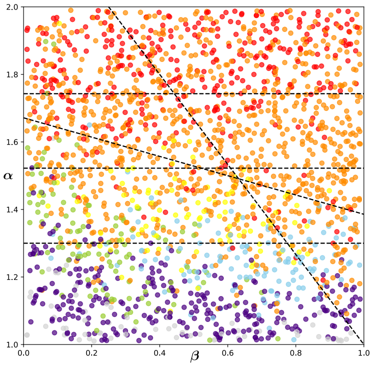

(d) Density-dependent growth, $n = 10^6$

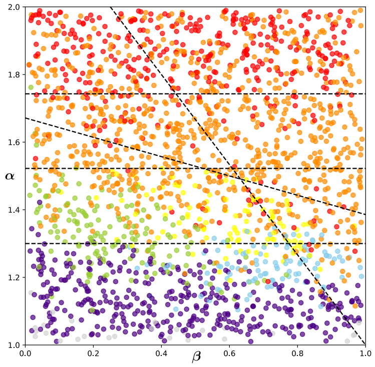

(e) Density-dependent growth, $n = 10^9$

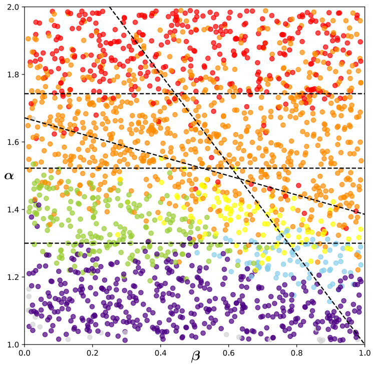

(f) Density-dependent growth, $n = 10^{12}$

Figure S2: Observed paths in simulations with exponential (a,b,c) and density-dependent (d,e,f) growth: each point corresponds to a simulation and is colored according to the observed scenario. For each subplot, 2,000 simulations were run for 7 cycles with $n = 10^6$ (a,d), $n = 10^9$ (b,e) or $n = 10^{12}$ (c,f). $\delta = 1.3$, $b_{00} = 0.3, b_{10} = 0.45, b_{01} = 1, b_{11} = 1.1$ and $d_{00} = d_{10} = d_{01} = d_{11} = 0.1$. For simulations with density-dependence, $p = 0.5$ and $s = 0.1$.

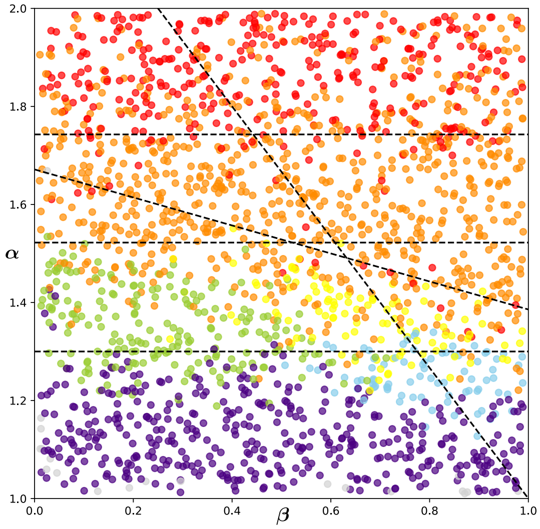

(a) 7 cycles, $p = 0.5$

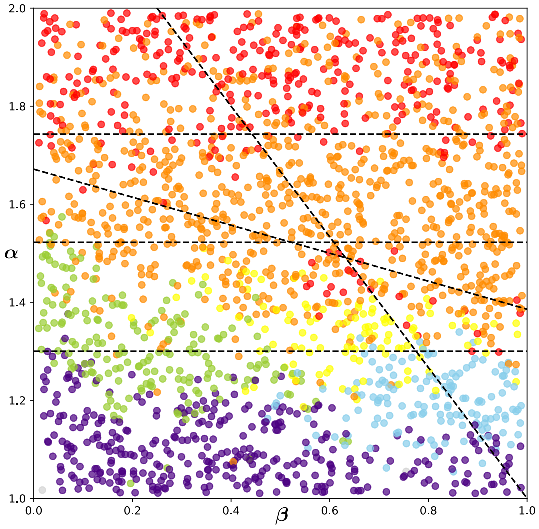

(b) 30 cycles, $p = 0.5$

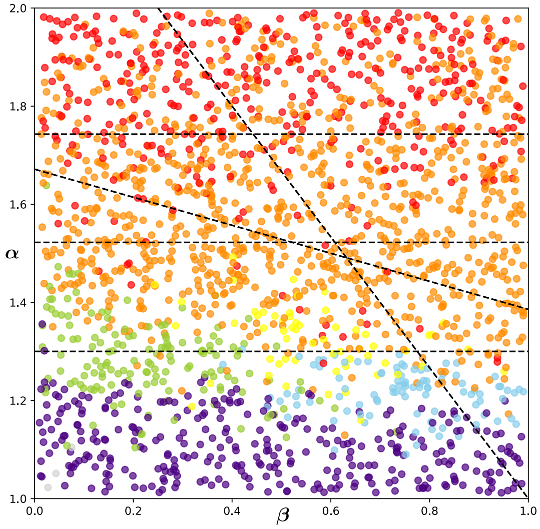

(c) 7 cycles, $p = 0.9$

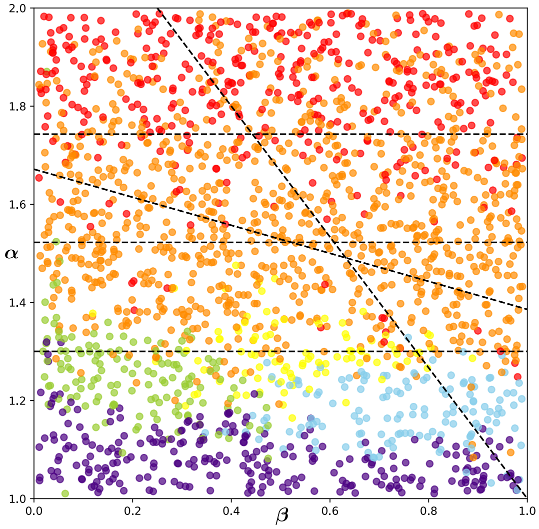

(d) 30 cycles, $p = 0.9$

Figure S3: Influence of density-dependence and the number of cycles: observed paths in simulations with density-dependent growth. Each point corresponds to a simulation and is colored according to the observed scenario. Simulations were run with $p = 0.5$ (a,b) or $p = 0.9$ (c,d), for 7 (a,c) or 30 (b,d) cycles. $n = 10^{12}$, $s = 0.1, \delta = 1.3, b_{00} = 0.3, b_{10} = 0.45, b_{01} = 1, b_{11} = 1.1$ and $d_{00} = d_{10} = d_{01} = d_{11} = 0.1$.

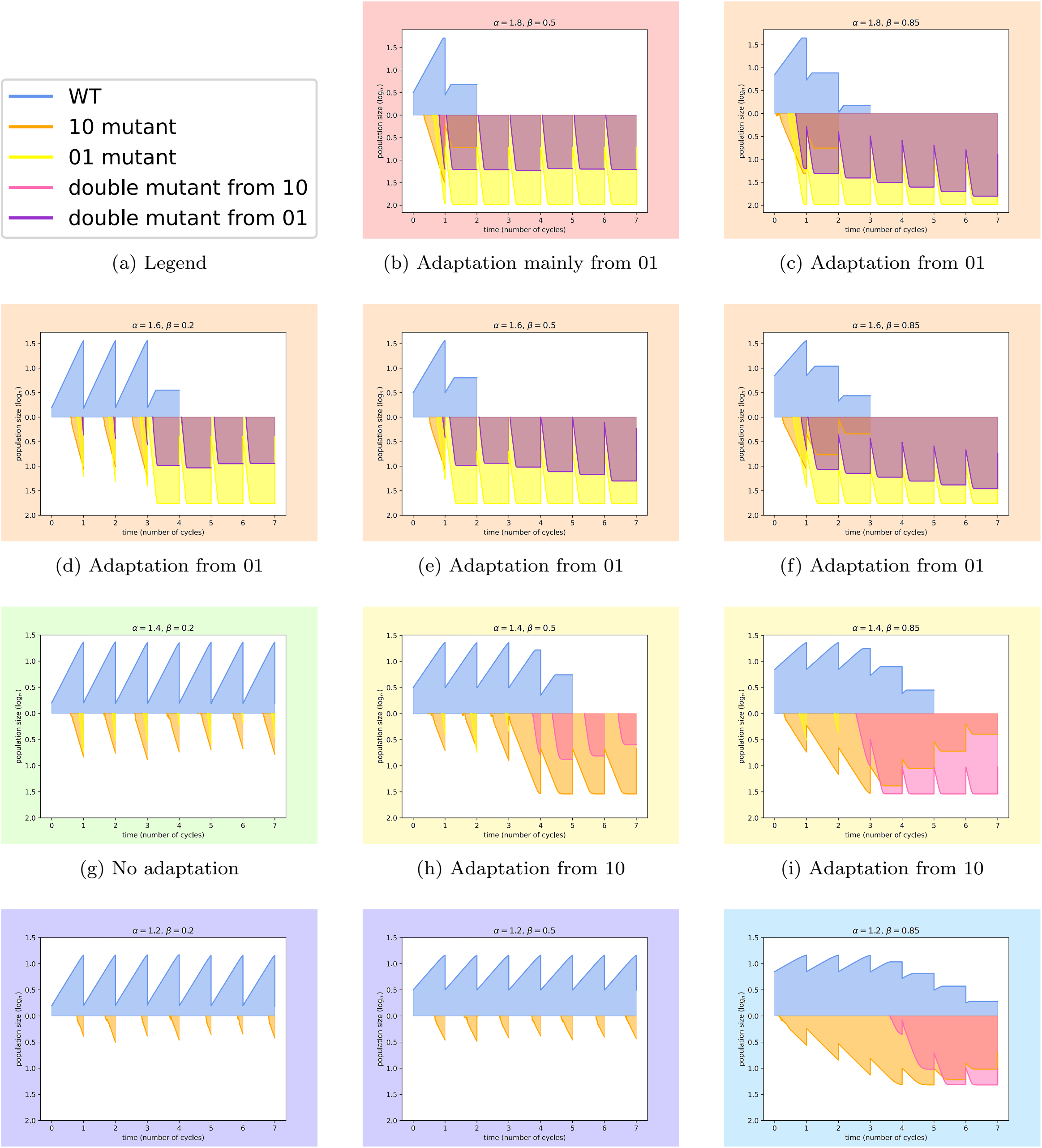

(a) Legend

(b) Adaptation mainly from 01

(c) Adaptation from 01

(d) Adaptation from 01

(e) Adaptation from 01

(f) Adaptation from 01

(g) No adaptation

(h) Adaptation from 10

(i) Adaptation from 10

(j) No adaptation

(k) No adaptation

(l) Adaptation from 10

Figure S4: Examples of simulated trajectories for $(\alpha, \beta) \in \{1.2, 1.4, 1.6, 1.8\} \times \{0.2, 0.5, 0.85\}$, with density-dependence. WT population size is above, mutant population sizes are plotted below and superimposed. Population sizes are plotted on $\log_n$ scale. Each simulation was run for 7 cycles, with parameters $n = 10^9$, $p = 0.5$, $s = 0.1$, $\delta = 1.3$, $b_{00} = 0.3$, $b_{10} = 0.45$, $b_{01} = 1$, $b_{11} = 1.1$ and $d_{00} = d_{10} = d_{01} = d_{11} = 0.1$.

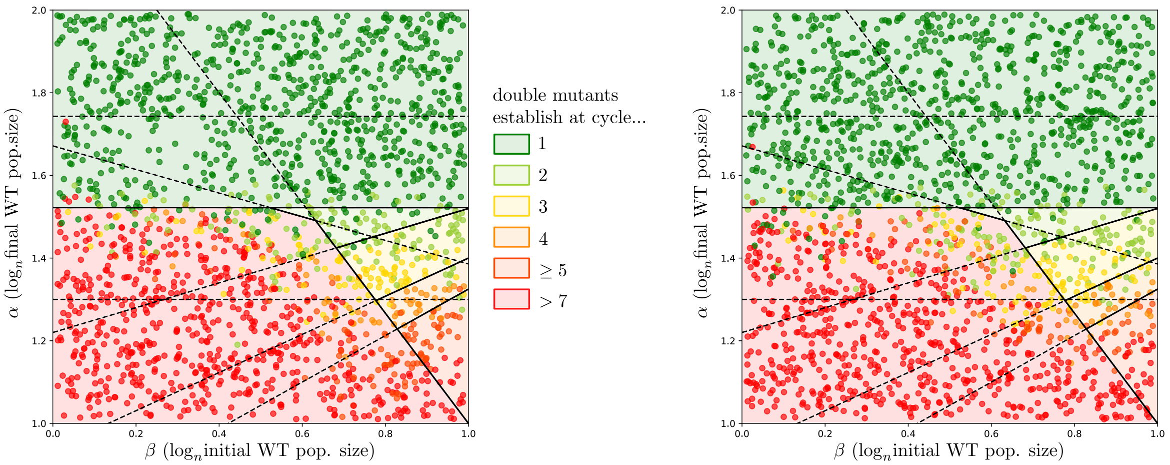

(a) Exponential growth

(b) Density-dependent growth

Figure S5: Observed cycles of double mutant emergence in 2,000 simulations with exponential growth (a) and density-dependent growth (b) for 7 cycles, with $n = 10^{12}$. $\delta = 1.3$, $b_{00} = 0.3, b_{10} = 0.45, b_{01} = 1, b_{11} = 1.1$ and $d_{00} = d_{10} = d_{01} = d_{11} = 0.1$. For simulations with density-dependence, $p = 0.5$ and $s = 0.1$. Point are colored according to the cycle at which double mutants established. Boundaries and background colors are theoretical predictions for exponential growth and large n.
